# Editorial: Advances in metabolic mechanisms of aging and its related diseases, Volume II

**DOI:** 10.3389/fphys.2023.1145165

**Published:** 2023-01-24

**Authors:** Daniele Lettieri-Barbato, Natascia Ventura, Raffaella Faraonio, Katia Aquilano

**Affiliations:** ^1^ Department of Biology, University of Rome Tor Vergata, Rome, Italy; ^2^ IRCCS Fondazione Santa Lucia, Rome, Italy; ^3^ IUF—Leibniz Research Institute for Environmental Medicine, Dusseldorf, Germany; ^4^ Medical Faculty, Institute of Clinical Chemistry and Laboratory Diagnostic, Heinrich Heine University of Düsseldorf, Düsseldorf, Germany; ^5^ Department of Molecular Medicine and Medical Biotechnology, University of Naples Federico II, Naples, Italy

**Keywords:** antioxidants, endoplasmic reticulum, insulin resistance, mitochondria, viral infection, neuregulin 4, osteoporosis

A well-organized framework of signaling pathways assures optimal cellular, organ and organism operation and is involved in DNA repair, replication and surveillance; recognition and removal of defective lipids and proteins as well as defective organelles and cells; and defense against pathogens and several types of injuries. Aging can be defined as the deterioration and failure of such functional properties. This condition leads to a loss of homeostasis and decreased adaptability to internal and environmental stress yielding in the elderly to an increased vulnerability to disease and mortality.

Metabolic alterations are considered hallmarks of aging and age-related diseases. Numerous metabolic pathways underlying the aging process are now emerging that are not exclusively linked to oxidative stress and/or mitochondrial dysfunction “*per se*” but rather to the failure of dynamic connections among signaling inputs triggered by the various cellular organelles. Changes in lipid metabolism occur during aging, lifespan and age-related disease (Johnson and Stolzing, 2019). Importantly, beside for sustaining proper protein folding and Ca^2+^ homeostasis, endoplasmic reticulum (ER) is a key organelle for maintaining lipid balance, particularly of glycerolipids and glycerophospholipids (Jacquemyn et al., 2017). Furthermore, dynamic interactions among ER, lipid droplets and mitochondria are essential for lipid homeostasis. In this Research Topic, Lourenço et al. provide, for the first time, data supporting a cross-talk between mitochondrial inner membrane prohibitin complex (PHB) and ER in the regulation of lipid metabolism and ageing. In their original article, in *C. elegans*, authors show that PHB modulates triacylglycerol and phospholipid pools at the young adult stage and during ageing depending on the genetic background of the worms. Specifically, PHB deficiency differently affects lifespan decreasing or increasing it in wild type or *daf-2* (encoding for insulin receptor) mutants, respectively. Lipid perturbation can boost an ER-unfolded protein response (UPR^ER^). Notably, authors showed that while PHB depletion induces UPR^ER^ in wild type worms, in *daf-2* mutants such UPR^ER^ is attenuated. Finally, the authors demonstrate that deficiency of DNJ-21, a co-chaperone interacting with PHB and implicated in cardiolipin remodeling, phenocopies the effects of PHB depletion on the UPR^ER^ stress response and on lifespan. These results highlight that ER homeostasis plays a critical role in aging signals and that dysregulation of the crosstalk between ER and mitochondria *via* PHB depletion, affects lipid metabolism and consequently longevity pathways.

Redox imbalance as well as mitochondrial dysfunction could represent crucial drivers of several physio-pathological events, including viral infections. In this context, redox and mitochondrial state affect the severity of infectious diseases of viral origin, including COVID-19 flu (Ren et al., 2020; Dominguez et al., 2021). In their study Grossini et al. aimed at finding any possible association between the plasmatic levels of oxidants/antioxidants, mitochondrial function, and the onset of COVID-19 in the elderly, in order to better clarify both the role of the redox state in old people and its possible implications to their susceptibility to COVID-19. They collected plasma of 60 elderly patients admitted to a long-term care/nursing homes and measured different parameters linked to oxidants/antioxidants conditions. They show that in old patients the redox state is quite balanced due to preserved antioxidant systems. By *in vitro* experiments, in which plasma taken from old subjects was used to treat HUVEC cells, authors demonstrate that mitochondrial membrane potential is preserved as well. By screening clinical records for symptoms suggestive of COVID-19, 37 possible cases of affected patients among 60 were found. Then, the study aimed to correlate the importance of mitochondrial function with patients’ outcomes. By measuring mitochondrial membrane potential of HUVEC cells upon treatments with baseline plasma, authors demonstrate that mitochondrial membrane potential can be *bona fide* considered a predictor of COVID-19 onset. These findings correlate the key role of dysfunctional mitochondria to aging and age-related diseases (Balaban et al., 2005) as well as to defects in immunological response upon viral infections (Ren et al., 2020; Shenoy, 2020) and this could be relevant in the context of COVID-19 syndrome.

Osteoporosis is a common age-related disease that weakens bones and is associated with alteration in body metabolism (Srivastava and Deal, 2002). This condition primarily occurs in women after menopause and in individuals of both sexes during aging (Pietschmann et al., 2009). Estrogen deficiency represents the major cause of osteoporosis in postmenopausal women, and this hormonal condition is associated with blunted bone homeostasis, with both low osteogenesis and enhanced osteoclastogenesis (Srivastava and Deal, 2002). Not surprisingly, it has been suggested that osteoporosis caused by estrogen deficiency leads to metabolic and oxidative stress-related dysfunctions (Foger-Samwald et al., 2022; Thapa et al., 2022); hence antioxidant defense systems could be important for prevention/treatment of postmenopausal osteoporosis. The review of Yang et al. brings together relevant data on the three classes of antioxidant defense systems in relation to the osteoporosis in postmenopausal women, namely antioxidant molecules, antioxidant enzymes, and repair enzymes. Firstly, they describe how estrogen deficiency boosts ROS production in both osteoblasts and osteoclasts and then discussed the importance of GSH/Nrf2 antioxidant pathway as one of the main components of osteogenesis and osteoclastogenesis balance. In this regard, GSH and vitamin C are discussed as molecules participating in lipid peroxide trapping mediated by vitamin E, which is found to alleviate osteoporosis. Increased metal levels, in particular iron, are key ROS inducers, thus endogenous antioxidant proteins like Ferritin and Ceruloplasmin are discussed as effective means in osteoporosis treatments. Other antioxidant enzymes (i.e., Superoxide oxidoreductase, Catalase, Glutathione peroxidase) are described as relevant in the pathophysiology of postmenopausal osteoporosis signals. Finally, in such a context, the relationship between repair enzymes of DNA damages and lipid peroxidation is summarized. The authors concluded that antioxidants ought to be explored for postmenopausal osteoporosis diseases.

Insulin resistance is another important hallmark of age-related obesity and increases the risk to develop type 2 diabetes (Taylor, 2012). Another interesting Brief Research Report by Martinez et al. looked at levels of serum Neuregulin 4 (NRG4) to evaluate its contribution in obesity-associated metabolic disturbances. NRG4 belongs to a family of related signaling proteins that mediate numerous effects by binding to ErbB receptors; however, its role in human obesity remains to be studied more in depth (Tutunchi et al., 2020). Here the authors measured circulating NRG4 levels in 89 participants and observed that NRG4 amounts correlated negatively with insulin sensitivity and positively with chronic inflammatory marker (hsCRP). Thus, they tested *in vitro* if human recombinant NRG4 administration on HepG2 cells affects expression of gluconeogenic- and mitochondrial biogenesis-related genes upon treatments with the fatty acid palmitate. The obtained results indicate that NRG4 impacts negatively on gluconeogenesis and mitochondrial respiration with no effects on lipid metabolism-related genes. This study also suggests that the beneficial effects observed in mice could be context-dependent and more investigations are required to assess if NRG4 may impact on human body metabolism.
